# Model‐informed drug repurposing: Viral kinetic modelling to prioritize rational drug combinations for COVID‐19

**DOI:** 10.1111/bcp.14486

**Published:** 2020-08-05

**Authors:** Michael G. Dodds, Rajesh Krishna, Antonio Goncalves, Craig R. Rayner

**Affiliations:** ^1^ Certara, USA Inc. Princeton NJ; ^2^ Université de Paris, IAME, INSERM Paris France; ^3^ Monash Institute of Pharmaceutical Sciences Monash University Melbourne Australia

**Keywords:** combination therapy, COVID‐19, modelling, repurposing, viral cell cycle

## Abstract

**Aim:**

We hypothesized that viral kinetic modelling could be helpful to prioritize rational drug combinations for COVID‐19. The aim of this research was to use a viral cell cycle model of SARS‐CoV‐2 to explore the potential impact drugs, or combinations of drugs, that act at different stages in the viral life cycle might have on various metrics of infection outcome relevant in the early stages of COVID‐19 disease.

**Methods:**

Using a target‐cell limited model structure that has been used to characterize viral load dynamics from COVID‐19 patients, we performed simulations to inform on the combinations of therapeutics targeting specific rate constants. The endpoints and metrics included viral load area under the curve (AUC), duration of viral shedding and epithelial cells infected. Based on the known kinetics of the SARS‐CoV‐2 life cycle, we rank ordered potential targeted approaches involving repurposed, low‐potency agents.

**Results:**

Our simulations suggest that targeting multiple points central to viral replication within infected host cells or release from those cells is a viable strategy for reducing both viral load and host cell infection. In addition, we observed that the time‐window opportunity for a therapeutic intervention to effect duration of viral shedding exceeds the effect on sparing epithelial cells from infection or impact on viral load AUC. Furthermore, the impact on reduction on duration of shedding may extend further in patients who exhibit a prolonged shedder phenotype.

**Conclusions:**

Our work highlights the use of model‐informed drug repurposing approaches to better rationalize effective treatments for COVID‐19.

1 **What is already known about this subject**

- Given the dearth of effective antiviral monotherapies for COVID‐19, the need to evaluate combinations, as outlined by the World Health Organization in the initial coronavirus roadmap, is being re‐emphasized. Significant inefficiencies in global clinical trials efforts for COVID‐19 have also resulted in calls for action from the clinical and quantitative pharmacology community to support streamlining of efforts, including using model informed approaches.
- Viral cell cycle kinetics models as well as their limitations have been well described in the literature, but their potential to inform combination treatment strategies for SARS‐CoV‐2 has not been described.
- The fundamental principles of simple viral cell cycle models remain largely conserved across different viruses even though the specific kinetics within the viral cell cycle may differ, therefore lessons learned from prior applications can be leveraged to inform rationale drug treatments for COVID‐19.

**What this study adds**

- This study postulates that a simple viral cell cycle model based on SARS‐CoV‐2 may serve as a framework to understand the impact of modulating specific rate constants as site of action for treatment interventions and proposes that effectiveness is directly related to these sites of action.
- Using a viral cell cycle model framework, individual and combination treatment choices, the time window of effective treatment and intervention strategies aligned to treatment goals are proposed for COVID‐19.

## 1 INTRODUCTION

The ongoing coronavirus pandemic has resulted in more than 7.4 million cases of infection and more than 418 000 deaths worldwide, including more than 115 000 deaths in the United States alone as of 11 June 2020.^1^, ^2^ The SARS‐CoV‐2 virus has been isolated and the disease designated as COVID‐19.^1^, ^3^, ^4^

According to the Siddiqui and Mehra typology of COVID‐19 disease progression, there are three distinct phases of infection.^5^ Stage 1 is mild disease starting immediately upon infection where SARS‐CoV‐2 multiplies and engages with the host respiratory system. Stage 2 of the clinical progression involves moderate pulmonary symptomology wherein infected subjects show early stages of viral pneumonia, with pronounced cough and fever. Stage 3 is the severe form of infection where there is evidence of systemic hyperinflammation. Here, systemic inflammation markers are elevated. Patients progress to shock, vasoplegia, respiratory failure and cardiopulmonary collapse. Stage 3 is associated with a poor prognosis.^5^

As of 11 June 2020, there were 1166 clinical interventional studies registered in clinicaltrials.gov with therapies targeting COVID‐19. The vast majority of these studies are not randomized placebo‐controlled clinical trials and they often included patients with COVID‐19 who were hospitalized and presented with the severe form, limiting the interpretation of results from a drug development perspective.^6^, ^7^, ^8^, ^9^ Significant inefficiencies in global clinical trials efforts for COVID‐19 have also resulted in calls for action from the clinical and quantitative pharmacology community to support streamlining of efforts, including using model informed approaches.^10^

**FIGURE 1:**
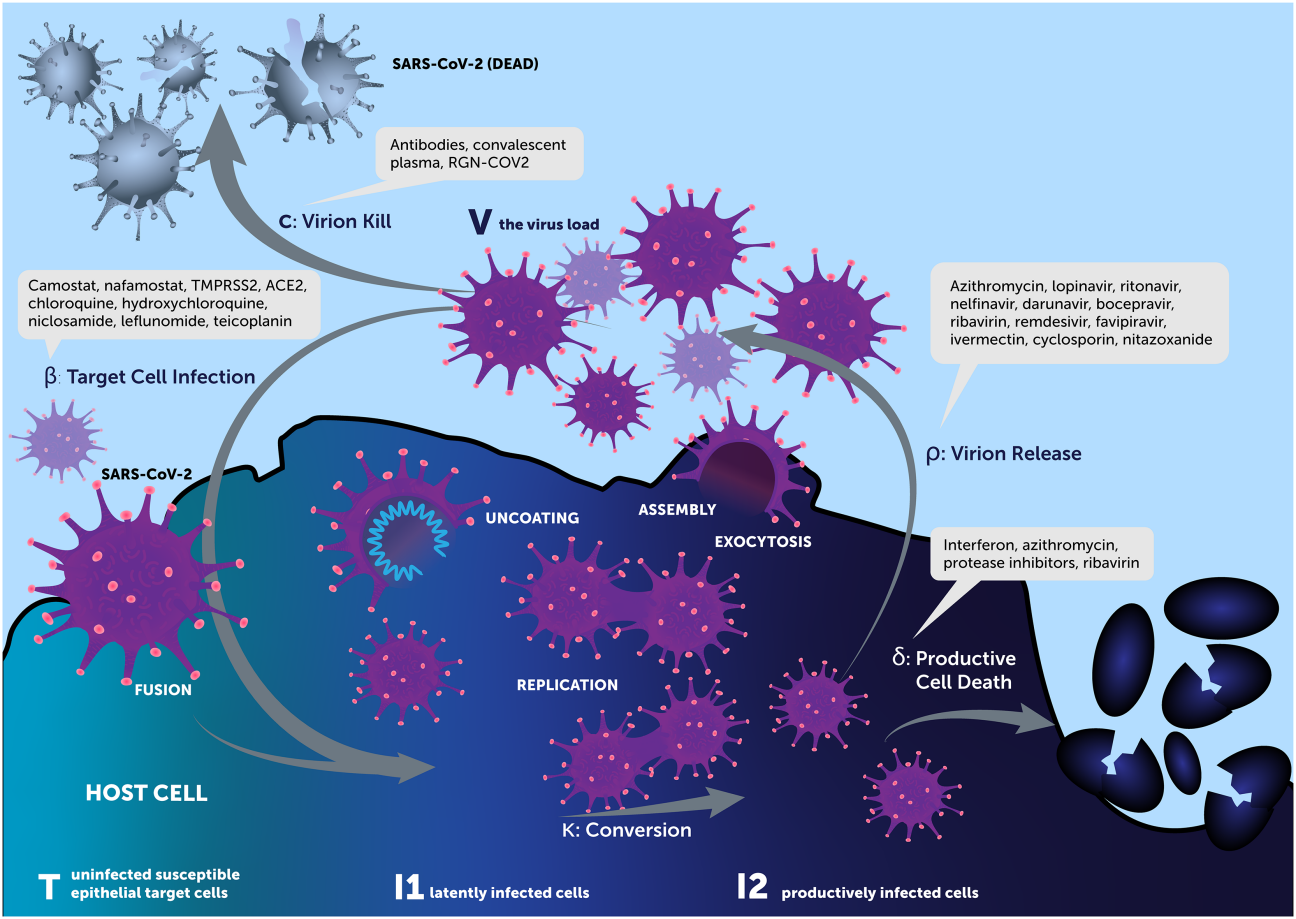
Cell cycle model and associated specific rate constants as target for therapeutics

Given the dearth of effective antiviral monotherapies for COVID‐19, the need to evaluate therapeutic combinations is being re‐emphasized, as was initially described in the World Health Organization's Novel Coronavirus Global Research and Innovation forum.^11^ We hypothesize that there are two key considerations for candidate antiviral therapeutic interventions, one being the timing of intervention relative to the infection stage, but more importantly where in the viral cell cycle these therapies interact. This cell cycle dependency of treatment options forms the central premise of our investigations and is further conceptualized in Figure 1.

Viral load dynamics has been elevated to surrogate status in the management of HIV by the United States Food and Drug Administration (FDA), and is aligned with clinical outcome for respiratory viral infections, including seasonal and emerging influenza strains in various populations,^12^ and correlated with clinical outcome in SARS‐CoV‐2 infection.^13^ Duration of viral shedding and impact of therapeutic interventions has been linked to transmission and health economic models, demonstrating indirect benefits of individual treatment to societal outcomes for pandemic influenza.^14^ Such endpoints have been of critical importance in informing procurement and deployment decisions for interventions within healthcare systems during outbreak scenarios. Viral kinetic modelling has also been extensively used to support drug development decisions in the respiratory virus space.^15^ The aim of this research was to leverage a model of SARS‐CoV‐2 infection to support prioritization of drug combinations based on how their mechanisms interacted in a simulation setting to improve various metrics of infection outcome.

## 2 MATERIALS AND METHODS

### 2.1 Model

We used a target‐cell limited model with an eclipse phase based on an analysis published by Goncalves et al^16^ that was used to characterize the viral load dynamics of 13 hospitalized COVID‐19 patients from frequent nasopharyngeal swabs.

In simple terms, the target‐cell limited model integrates four entities, uninfected susceptible epithelial target cells (*T*), latently infected cells (*I1*), productively infected cells (*I2*) and the virus load (*V*), and is described by a system of nonlinear ordinary differential equations.^17^ Given the timescale of the infection, the model neglects target cell proliferation and natural death, and focuses on the process of epithelial cell depletion (*T*) by virus infection. When a virus (*V*) interacts with an uninfected target cell (*T*) at a defined infection rate, *β*, the target cells will become infected (*I1*) and remain so during an incubation period. These cells, in turn, convert to productively infected cells (*I2*) at a rate *k.* These cells then produce new virions (*V*) with a defined production rate *ρ.* Simultaneously, productively infected cells die at a certain rate *δ.* Circulating virions (*V*) are then cleared at a certain rate *c* from the body or go on to infect new cells as above. Based on the dynamics of the cell model and the associated mechanisms of actions of the currently experimented drugs for SARS‐CoV‐2 infection, we classify treatments to potentially affect one or more of the five different distinct check points in this model: *β*, *k*, *ρ*, *δ* and *c* (Figure 1).

We describe a model‐informed analytical framework that yields predictions on the most viable combinations of drugs matched by phase of clinical progression. For more details, readers are directed to the estimation publication^16^ for details on the analysis, assumptions and data. Briefly, key assumptions and limitations noted by the authors were assumption of most‐plausible incubation period of 5 days, model averaging to address model uncertainty, estimation of lumped parameters to address well‐known identifiability issues, and sensitivity analyses performed to assess model consistency across a range of assumed parameters. The authors performed a similar analysis presented here, focusing on specific drug effects and intervention time.

Specific model parameters for this exercise were *V*_0_ = 0.1 #/mL, *T*_0_ = 1.33E^5^ #/mL, *k* = 3 1/d, *δ* = 0.60 1/d, *β* = 2.21E‐5 mL/#/day, *ρ* = 22.7 1/d and *c* = 10 1/d. The parameters are consensus values from the model averaging process described by the authors. For state variables representing cells and virions, the meaning of “#” is “cells” and “copies”, respectively. The parameter *β* was derived from the reported *R*_0_ of 8.6 with the equation *β* = *R*_0_ × *δ* × *c*/[*T*_0_ × (*ρ* – *R*_0_ × *δ*)].

### 2.2 Intervention effects

Interventions were posited for the targets in the viral life cycle given in Figure 1. Intervention effects were modelled as inhibitory functions for *β*, *k* and *ρ* (eg, *β* × [1 – *I*_max_(*t*)]) and stimulatory functions for *δ* and *c* (eg, *δ* × [1 + *S*_max_(*t*)]). *S*_max_(*t*) and *I*_max_(*t*) were treated as step (Heaviside) functions with onset at times relative to the approximate viral peak, estimated as 9 days post infection: −6, −3, 0, +3 and +6 days. Intervention at viral peak −6 and −3 days represents cases of pre‐ and post‐exposure prophylaxis, intervention at 0 and +3 days represents cases of symptomatic presentation and intervention at +6 days represents cases of advanced infection.

Specific values of inhibition (*I*_max_) and stimulation (*S*_max_) were selected with the intention to “blanket” the space of pharmaceutical intervention from low to very high potency. Supporting Information Figure S1 reports the specific values, noting that the choices are interconvertible and can be expressed in terms of drug effect, *I*_max_ or *S*_max_:

$$\log10\left(\text{drug effect}\right)=\log10\left(S_{\max}+1\right)=–\log10\left(1–I_{\max}\right)$$

With this particular formation, a fair assessment of, for example, 1 log10 change in an inhibitory versus stimulatory effect can be made. Additionally, the specific values of individual effects (0, 0.333, 0.667, 1, 1.33, 1.67, 2 log10 change) can be summed for easy comparison. For example, a single effect with 1 log10 change (90% inhibition or 9‐fold stimulation) can be compared to an intervention with three effects each with 0.333 log10 change (53.6% inhibition or 1.15‐fold simulation each) fairly. If the three effects are strictly additive, then the total effect is 100 × (1 – [1 – 0.536]^3^) = 90% and would result in the same effect as a monotherapy effect of 90% if the effect is additive and targets the same pathway. In these simulations, different pathways are explicitly targeted and the model is nonlinear (second order) in the dynamics. Thus, difference in simulation outcome for two interventions with the same summed log10 change effect describe the synergy and anergy of targeting different pathways.

### 2.3 Simulations

Simulations were conducted in R (3.6.1) using the *RxODE* (0.9.2) package for numerical integration and the *tidyverse* (1.2.1) family of packages. An R script reproducing these results is provided in Supporting Information Data S1.

Each of the five checkpoints (*β*, *k*, *ρ*, *δ*, *c*) was probed with seven drug effect levels (0, 0.333, 0.667, 1, 1.33, 1.67, 2 log10 change) for a total of 16 797 simulation conditions. Each condition was replicated over five intervention times (viral peak at 9‐6, −3, 0, +3, +6 days) for a total of 84 035 condition‐times. However, simulations were reduced to cases with summed intervention effect between 0.333 and 2 log10 change, reducing the total simulations to 2310 intervention conditions.

### 2.4 Endpoints and metrics

We generated three key metrics for each simulation case.

#### 2.4.1 Viral load area under the curve

The viral load area under the curve (AUC) is calculated as the area under the viral load, *V*, time curve (copies/mL*day). This metric summarizes the total exposure to SARS‐CoV‐2 virions. This endpoint may have prognostic correlation to clinical outcome, but because it is highly dependent on the pre‐intervention viral load values it is insensitive to treatment interventions that occur beyond the peak viral load time. Moreover, because pre‐intervention loads are seldom measured experimentally, it remains a theoretical phase that provides important insights into pre (PrEP) and post exposure prophylaxis (PEP) strategies.

#### 2.4.2 Duration of viral shedding

The duration of time (days) for which the virus concentration, *V*, exceeds 100 copies/mL is often the lower limit of detection for qPCR assays for SARS‐CoV‐2.^18^ This duration of viral shedding metric summarizes the amount of time virus is detectable, which is considered a correlate with the time a patient is infectious. Reducing the duration of detectable virus thereby impacts transmission dynamics and risk to others within a population, but also may impact on the duration of isolation, containment or hospitalization of a patient, even if it does not correlate directly with an individual patient's signs and symptoms.

#### 2.4.3 Epithelial cells infected

*T* represents the number of epithelial target cells that are infected (cells/mL) by virus. This metric summarizes the damage to host lung epithelial host cells during the course of infection and is considered a proxy for the degree of pulmonary inflammatory response and lung tissue damage, and hence pulmonary clinical signs and symptoms within an individual patient. There is increasing evidence that other tissues and organs may be infected by SARS‐CoV‐2, but this is beyond the scope of this model.

These endpoints were compared to the reference case of the natural history, and expressed as the metric:

$$\log10\left(\text{treatment metric}/\mathrm{no}-\text{treatment metric}\right)$$

Therefore, a difference of 1 unit of a metric between two treatments indicates an order of magnitude change (ie, a decibel scale).

## 3 RESULTS

**FIGURE 2:**
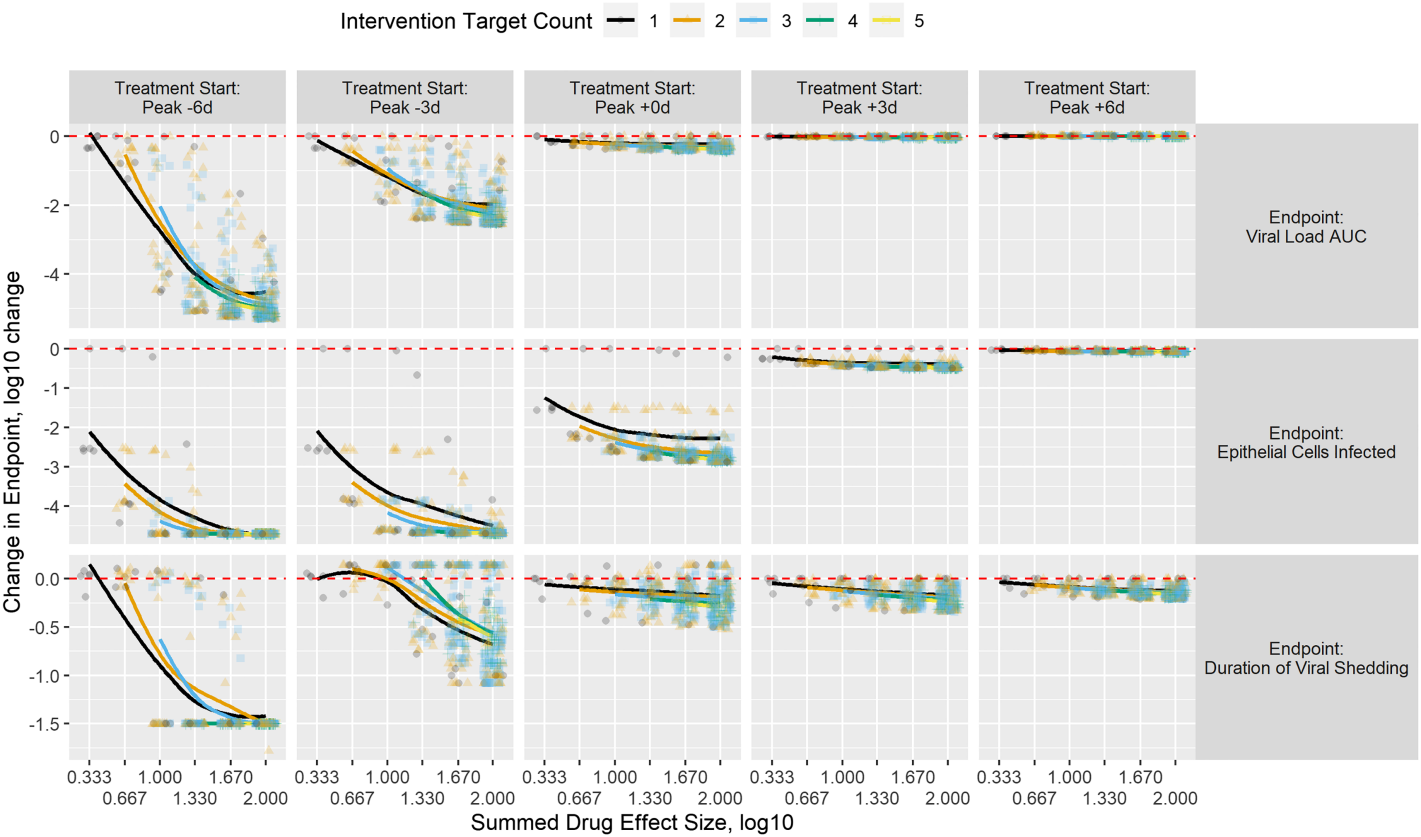
Change in endpoint metrics by treatment start, number of targets in the treatment and summed target effect in the treatment. Treatment initiation is shown by column relative to expected viral load peak. Endpoints are shown by row and are viral load AUC, epithelial cells infected and duration of viral shedding (levels ≥ 100 copies/mL). Change in endpoint relative to the no‐treatment control is reported on the *y* axis as log10(treatment outcome metric/no‐treatment outcome metric) with negative values representing improvements relative to the no‐treatment control. The number of targets in each intervention is shown by colour and shape. Each intervention is plotted by its summed drug effect on the *x* axis with some jittering in the *x* dimension to aid with overplotting issues. A LOESS (locally weighted smoothing) line is added per count of targets to show a general trend of improvement with increasing summed drug effect

An overview of treatment effects by intervention time and disease metric is summarized in Figure 2. The major observations from this overview are as follows. Outcome improves for every disease metric with earlier intervention, suggesting that PrEP and PEP provide the best opportunity for repurposed drugs with (potentially) low potency to impact disease. Treatments initiated after viral peak have little to no impact on the viral load AUC. Treatments initiated 3 days after viral peak have no impact on epithelial cells infected. Treatments initiated after the viral peak still have the potential to shorten the duration of viral shedding. Combinations of treatments targeting multiple pathways can be as effective or more effective as targeting single pathways with equivalent summed treatment effects. The heterogeneity observed at each summed effect level suggests that some combinations are less effective than others.

**FIGURE 3:**
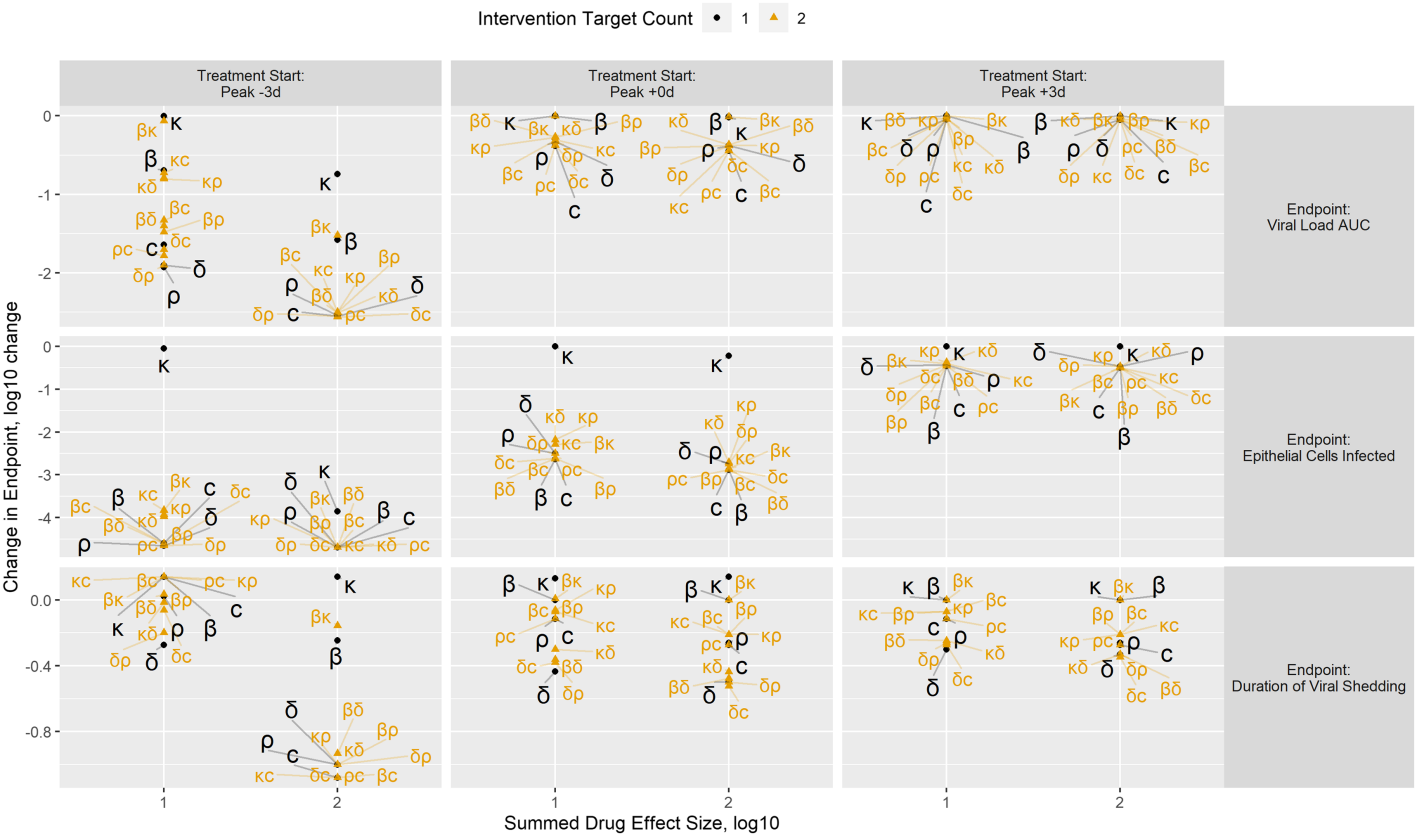
Comparisons of all one‐ and two‐target treatments by treatment initiation time, endpoint and summed drug effect. Treatment initiation is shown by column relative to expected viral load peak. Endpoints are shown by row. Change in endpoint relative to the no‐treatment control is reported on the *y* axis as log10(treatment outcome metric/no‐treatment outcome metric) with negative values representing improvements relative to the no‐treatment control. The number of targets in each intervention is shown by colour, size and shape. Each intervention is plotted by its summed drug effect on the *x* axis. Labels of the treatment targets appear with lines to connect them to the specific point on the diagram

To provide greater specificity on the relative importance of viral cell cycle pathways as intervention targets, comparisons of all one‐ and two‐target treatments by treatment initiation time (peak −3, 0, +3 days), endpoint, and 1 and 2 log10 summed drug effect are shown in Figure 3 **.** Results for all combinations are provided in Supporting Information Figure S2. The major observations from these simulations are as follows. Irrespective of viral cell cycle pathway, all endpoints are improved with earlier intervention. No endpoints are improved with single or combination treatments targeting *k* except in the cases of early (peak −3) intervention with potent (2 log10 summed) drug effect. That is, treatments only *delaying* the transition time between cell infection and production of new virions are not generally effective. Viral load AUC is improved with single or combination treatments targeting *c*; *δ* and *ρ* are interchangeable and modestly effective. Single or combination treatments involving *β* are not effective at or after viral peak. Duration of viral shedding is improved with single or combination treatments targeting *δ*; *ρ* and *c* are interchangeable and modestly effective. Single or combination treatments involving *β* are not effective at or after viral peak. Epithelial cells infected is improved with single or combination treatments targeting *β* or *c* which are interchangeable; *δ* and *ρ* are interchangeable and modestly effective.

A qualitative ranking of target choices by metric of interest is provided in Table 1. Slowing the transition, *k*, of infected epithelial cells from eclipse, *I1*, to productive, *I2*, is not effective relative to other target choices. Increasing turnover, *δ*, and/or decreasing productivity, *ρ*, of infected epithelial cells, *I2*, is predicted to have positive benefit for all metrics and should be considered the backbone of proposed combinations. Broadly speaking, targeting *δ* and *ρ* seeks to disrupt the production machinery of SARS‐CoV‐2. Increasing virion kill, *c*, to deplete extracellular virions, *V*, is predicted to have positive benefit for all metrics. Inhibiting infection, *β*, is interchangeable with *c* for the epithelial cells infected metric, but not the viral load AUC and duration of viral shedding: *c* both removes virions and prevents infection but *β* only prevents infection.

**TABLE 1:** Ranking and priority of endpoint changes by treatment target

Endpoint	*k* Delay productivity	*β* Inhibit infection	*c* Promote virion kill	*δ* Promote infected cell death	*ρ* Inhibit virion release
Viral load AUC (days*cells/mL)	−	−	++	+	+
Duration of viral shedding (days)	−	−	+	++	+
Epithelial cells infected (cells/mL)	−	++	++	+	+

Results are given as qualitative rankings: –, little effect/avoid; +, good effect/consider; ++, best effect/prioritize.

**FIGURE 4:**
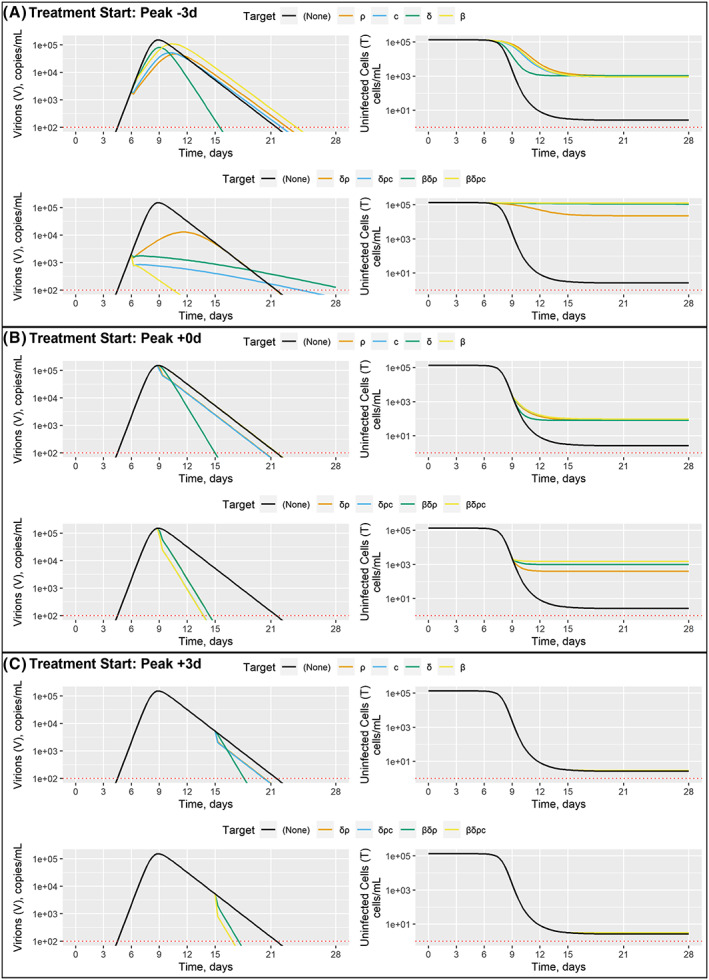
Example combination treatments. Interventions initiated 3 days before, at, and 3 days after peak viral count are shown panels A, B and C, respectively; within each panel, single‐target and multiple‐target interventions are shown in the top and bottom rows, respectively, virion and uninfected endpoint are shown by column, simulation values over time are reported on the *y* and *x* axes, respectively, and specific targets are shown by colour with the no‐intervention (negative control) case shown in black

Table 2 summarizes the association of the mechanism of action of currently tested repurposed molecules for COVID‐19 with the specific rate constants. To illustrate the potential benefit of combinations of repurposed drugs, example combinations were drawn from the current trial literature and are presented in Table 3.We assumed modest effect (0.333 log10 effect, 53.6% inhibition, 1.15 fold increase) for each target. Single‐target interventions were selected as *β*, *δ*, *ρ* and *c*, two‐target intervention was selected as *δρ*, three‐target interventions were selected as *δρc* and *βδρ*, and a four‐target intervention was selected as *βδρc.* Figure 4 shows the output of these simulations at selected intervention times. Supporting Information Figure S3 and Figure 4 report the predicted impact viral and infected epithelial cell kinetics assuming intervention 6 days before and 6 days after peak viral load, respectively. Figure 4A shows the predicted impact on viral and infected epithelial cell kinetics assuming intervention 3 days before peak viral load. For the single interventions (top row) *β*, *δ*, *ρ* and *c*, no single intervention is sufficient to halt viral growth, but each blunts the peak and may shift the timing of peak viral load. However, a meaningful (>2 log10) improvement in epithelial cells infected is expected. The combination interventions (bottom row) *δρ*, *βδρ*, *cδρ* and *βδρc* follow. The two‐target intervention *δρ* shows a similar “blunting and delaying” quality on viral load as the single‐target interventions, but better overall suppression of viral load and epithelial cell infection. In contrast, the three‐ and four‐target interventions halt viral growth and (nearly) abolish epithelial cell infection. As a reminder, each element of each intervention is assumed to have a modest effect, so the results shown for the multiple‐target combinations express their combined effect on viral load and infected epithelial cells.

**TABLE 2:** Association of the mechanism of action of the currently tested drugs with the cell cycle components

Cell cycle phase	Mechanism	Example drugs	References
*β*, target cell (infection) Rate at which circulating virions convert healthy epithelial cells to “eclipse” (nonproductive or pre‐productive) phase infected cells	Attachment phase	Camostat and nafamostat to TMPRSS2 chloroquine to ACE2	19, 20, 21
	Endosomal acidification	Chloroquine and hydroxychloroquine, niclosamide	19, 22, 23
	Cytoplasmatic assembly	Leflunomide	24
	Cleavage of viral spike protein by cathepsin L	Teicoplanin	25
*δ*, productive cell (death) Rate at which productively infected cells die	Programmed cell death, apoptosis, stress response	Interferon azithromycin	26, 27, 28
	Apoptosis	Protease inhibitors, ribavirin	29, 30
*p*, virion (release) Rate at which productively infected cells produce new virions	Unknown	Combination treatment (interferons and protease inhibitors) azithromycin	31, 32
	Viral proteolysis	Lopinavir, ritonavir, nelfinavir, darunavir, bocepravir, ribavirin	33, 34
	RNA‐dependent RNA polymerase	Remdesivir, favipiravir	34, 35
	Nonspecific	Ivermectin, cyclosporin, nitazoxanide azithromycin	32, 36, 37, 38
*c*, virion (kill)	Unknown	Antibodies, convalescent plasma, RGN‐COV2	39

**TABLE 3:** Example combinations focusing on host cell impact and virion killing

Viral cell cycle	Example regimens	Comment
*p*, virion (release)	Remdesivir alone^8^	RCT of intravenous remdesivir in adults hospitalized with evidence of lower respiratory tract involvement, showed improved median recovery time of 11 *vs* 15 days
*c*, virion (kill)	Antibodies, convalescent plasma, vaccines	Under investigation
*δ*, productive cell (death) *p*, virion (release)	Interferon‐b‐1b, ribavirin^9^ Protease inhibitors	Open label, prospective, randomized early treatment study (median 5 days, [IQR 3‐7 days] since symptom onset) showed triple combination reduced viral shedding by 5 days sooner *vs* lop/r alone
*β*, target cell (infection) *δ*, productive cell (death) *p*, virion (release)	Hydroxychloroquine Azithromycin	Small case series with multiple issues with trial design and no comparator group limit inferences on virologic or clinical efficacy^6^ Concerns on QT prolongation^40^
	Remdesivir + hydroxycloroquine + lopinavir/ritonavir and/or interferon	Potential RDV triple or quad combination antiviral regimen that covers three parts of viral cell cycle

The predicted impact on viral and infected epithelial cell kinetics assuming intervention at peak viral load is shown in Figure 4B. As above, the single interventions have modest effect on viral load, with *δ* identified as ideal for reducing duration of viral shedding and *c* identified as ideal for reducing viral load AUC (Table 1). A 3 log10 reduction in uninfected epithelial cells is expected. The three‐ and four‐target interventions somewhat improve duration of viral shedding, but the biggest gain is observed in a 1 log10 improvement in epithelial cells infected, with *β* identified as ideal (Table 1).

Figure 4C shows the predicted impact on viral and infected epithelial cell kinetics assuming intervention 3 days before peak viral load. Some modest gains are possible for duration of viral shedding, with *δ* identified as ideal. Treatments targeting *c* are identified as ideal for reducing viral load AUC (Table 1). Very little improvement in epithelial cells infected is predicted, reinforcing the primary finding of this work and others that early intervention is critical. Here, we explicitly report the effect on host cell damage, which has been underappreciated in prior efforts.

## 4 DISCUSSION

We have argued that the selectivity of antiviral therapy can be significantly enhanced by exploiting matching of the drug based on its purported mechanism of action with viral cell cycle dynamics. Table 2 summarizes the association of the mechanism of action of currently tested repurposed molecules for COVID‐19 with specific rate constants. It is interesting to note that of these drugs, those drugs that target the conversion rate constant alone, such as those that target viral proteolysis, RNA‐dependent RNA polymerase, and those that act nonspecifically, such as ivermectin, cyclosporine and nitazoxanide, are least likely to result in meaningful efficacy based on the model described in this manuscript. This is supported by the weight of evidence (clinical trial or white paper based arguments) that has been generated so far on remdesivir,^7^, ^8^ protease inhibitors,^41^ and ivermectin,^42^ which indicates that the effects are likely to be negligible to modest at best.

There are various population‐level viral cell cycle models available in the literature. These vary by complexity of the models, ranging from the most parsimonious target‐cell limited model to the most sophisticated yet complex multiscale models that describe virus‐host interactions.^43^, ^44^, ^45^ These models were used to characterize a variety of viruses, including HIV, hepatitis C virus (HCV) and influenza A. It is important to acknowledge that all available modelling methods of viral life cycle have limitations, with the most sophisticated models being particularly data hungry and as model complexity increases, so too does the inherent challenge of parameter cross‐correlation and identifiability during the parameter estimation process. Nguyen et al^46^ have concluded that whilst accurate estimation of biological parameters is inherently problematic, if not impossible, the “above issues are not *defacto* worrisome barriers if the primary purpose is to evaluate working hypotheses”, which is the focus of this study. On balance, we elected to use a previously published target‐cell limited model with an eclipse phase^16^ that had characterized the viral load dynamics of SARS‐CoV‐2 from infected patients as the basis for a framework to test the impact of the currently envisaged antiviral armamentarium. The estimation manuscript^16^ that underpins the simulations presented here went to considerable lengths to address the limitations of the viral model and the paucity of available data. In particular, the assumption of the most plausible incubation period of 5 days was documented. The authors performed model averaging procedures to address model uncertainty. Where necessary, the authors estimated lumped parameters to address well‐known identifiability issues with target‐cell limited viral models. Finally, sensitivity analyses were performed to assess model consistency across a range of assumed parameters that could not be estimated from the available data.

The authors would strongly encourage the hypotheses proposed from this framework to be tested in preclinical and clinical investigations. Furthermore, as new data is generated, model insights are validated and/or models evolve, the authors would also recommend expanding this framework to a fuller quantitative and systems pharmacology (QSP) model which might provide a scaffold for the generation of testable hypotheses incorporating interventions impacting downstream host‐inflammatory pathways. We consider our efforts as a parsimonious first module to inform and inspire more comprehensive QSP strategies, not only for COVID‐19 but for emerging viruses in general.

Our simulations demonstrated some important themes for consideration of combination treatments targeting the SARS‐CoV‐2 life cycle. In general, antivirals should be initiated as early in the course of infection as possible to maximize impact on viral load AUC, duration of viral shedding and epithelial cells infected. This was a common theme across the scenarios and endpoints evaluated. According to our simulations, beginning treatment beyond 3 days after peak viral load is unlikely to have any meaningful impact on epithelial cells infected, which may correlate with patient symptoms, but benefit for later intervention may persist beyond 3 days for duration of viral shedding, which may correlated with clinical and public health endpoints. As prolonged viral shedding phenotypes are described for influenza^47^ and COVID‐19,^48^ we performed sensitivity analyses with *c* and *δ.* We observed in prolonged viral shedder phenotypes that cessation of viral shedding benefit persists for therapeutics that promote virion kill (*c*) and infected cell death (*δ*) and inhibit virion release (*ρ*). Such interventions would be preferred to address so‐called SARS‐CoV‐2 super spreaders.^49^, ^50^

The primary outcome of this simulation study is captured in Table 1 **,** demonstrating that interventions targeting the host‐cell “factories” for de novo virions are broadly effective in reducing viral load AUC, epithelial cells infected and duration of viral shedding. Mechanisms that promote infected cell death (*δ*) and/or reduce copies of virions per infection (*ρ*) achieve these goals. Simulations results suggest interchangeability of these effects, with the noted exception of reducing duration of viral shedding where *δ* is superior to *ρ.* Interchangeability suggests additive effects, from a simplistic anergy/additivity/synergy perspective, but also offers an opportunity to combine two low‐potency agents, each targeting one mechanism, to boost the overall potency of the combination. Within the host cells, simply delaying the viral replication machinery (*k*) is not a good strategy.

Outside or on the border of host cells, Table 1 reports differing strategies that depend on the objective of the intervention. Mechanisms that remove circulating virus (*c*) are broadly effective in reducing the viral load AUC, epithelial cells infected and duration of viral shedding. Mechanisms that prevent viral entry and infection of host cells (*β*) are only effective prior to peak viral load or if only focused on sparing host cell infection (epithelial cells infected). Put simply, killing virus (*c*) both removes virus and prevents infection. Vaccines and antibodies fall into the category of removing circulating virus (*c*), and are predicted to have strong effects even at low potency if administered early (or before, in the case of vaccines) in the course of infection.

Given that the full‐time course is rarely measured in clinical evaluations with the exception of PEP studies, viral load AUC is a largely insensitive endpoint to evaluate potential therapeutic interventions. This is because of their dependence on the pre‐intervention viral load values. For a therapeutic intervention to work in this regard, it needs to exhibit a rapid pharmacological onset (eg, loading dose, direct rather than indirect pharmacology) and needs to be effective at clearing the virus. Treatments targeting *c* (killing of released virions) were the most effective, meaning that interventions like convalescent plasma or investigational antibodies would be anticipated to be most likely to impact total viral load meaningfully.

Of the therapies being investigated, remdesivir was recently shown in hospitalized adults with moderate disease to provide a 31% faster time to recovery than those who received placebo (*P* < 0.001),^8^ but no virologic information was reported. However, in the study by Wang et al,^7^ remdesivir had no meaningful effect on viral load. Remdesivir is thought to play a role in the incorporation into new viral RNA, leading to the inability of the viral polymerase to add new RNA. In the absence of key mechanistic information, we assumed that remdesivir reduces the production of new virions by halting replication of its genome, and thus its effect is proximally associated with *ρ.* It is possible that remdesivir may show meaningful efficacy if studied in an earlier infection phase. Future data on remdesivir in early onset mild patients with COVID‐19, combined with suitable therapeutics, will likely inform on the benefits of early intervention for this molecule.

Duration of viral shedding is less time sensitive to perturbation than viral load AUC and epithelial cells infected, and is also influenced by a broader array of pharmacological interventions in the SARS‐CoV‐2 life cycle. Unlike both epithelial cells infected and viral load AUC, treatments targeting *δ* (death of infected cells) were the most effective against duration of viral shedding. The concept of early intervention with combination treatments targeting *δ* was validated in the clinic recently, where an open label, prospective, randomized early treatment study (median 5 days, [IQR 3‐7 days] since symptom onset) showed a triple combination of ribavirin, lopinavir/ritonavir and interferon (which all target *δρ*) reduced viral shedding by 5 days sooner versus lopinavir/ritonavir alone.^9^ Such findings may translate into meaningful benefits for patients and society, as duration of viral shedding may impact duration of hospital stay or isolation for an individual, and risk of transmission to others and the associated costs from a public health perspective.^14^

The SARS‐CoV‐2 life cycle provides some foundational basis for the selection of existing treatments with pharmacological plausibility within a set of combination regimens. To minimize epithelial cells infected and thus potential consequences of downstream cytotoxicity and pulmonary inflammation, a treatment regimen should include currently available treatment(s) that maximize pharmacology on *β* (inhibition of new epithelial cell infection) like camostat, chloroquine and hydroxychloroquine, or influence *ρ* as evidenced via remdesivir. To reduce duration of viral shedding, treatment regimens should include components that effectively reduce *δ* (death of infected cells), such as ribavirin, lopinavir/ritonavir and/or interferon. Experimental interventions like convalescent plasma, or investigational antibodies such as RGN‐COV2 and other investigational antibody treatments targeting *c* (killing of released virions) have the most significant promise in rapidly reducing viral load and sparing epithelial cells from infection and will be a welcome addition to the combination armamentarium.

## 5 FUTURE DIRECTIONS

Our model‐informed analysis underscores the need to include the key features of the viral cell cycle from the perspective of dynamic models to leverage the significance of cell‐cycle checkpoints (vis à vis specific rate constants) for emerging therapeutics. Our model builds upon previously described models by extending their utility into assessment of the value of combinations. Such an approach will be invaluable for clinicians and trialists to develop informed hypotheses based on cell‐cycle selectivity and specificity. The fundamental premise for this approach assumes that cell kinetics and durability of response are intricately regulated and can only be disrupted by a drug that has the specificity for that particular phase.

The model leveraged here is parsimonious and offers a quick, reliable method to triage therapeutics entering clinical assessment for the ongoing pandemic. Efforts are ongoing to further integrate wet‐lab inputs on the virus characterization model, including replication dynamics, tropism and cell culture susceptibility, but also integrating with drug characterization (including absorption, distribution, metabolism and elimination (ADME) profiles) and emerging clinical data from ongoing studies. We hope that further refinements as well as extension to broader incorporation of the downstream host inflammatory response and associated interventions such as immunomodulators, including IL‐6 inhibitors, will provide a comprehensive disease model backbone that could be fungible for inputing emerging virus pathogens. We believe that a comprehensive quantitative and systems pharmacology approach linking to wet‐lab data for emerging viruses can provide a structured scientific backbone that could revolutionize and rationalize our approach to selecting therapeutic interventions for future pandemics.

## 6 CONCLUSIONS

The simulation work presented here leverages the known kinetics of the SARS‐CoV‐2 life cycle to rank potential targeted approaches with a focus on the likely need to combine repurposed, low‐potency agents. These simulations suggest early intervention is critical and targeting multiple points important to viral replication within and release from infected host cells is a good strategy for reducing both viral load and host cell infection. In addition, we observed that the time‐window opportunity for a therapeutic intervention to effect duration of viral shedding exceeds the effect on sparing epithelial cells from infection or impact on viral load AUC. Furthermore, the impact on reduction on duration of shedding may extend further in patients who exhibit a prolonged shedder phenotype. Moreover, our model‐informed analyses directly address the call to action from clinical pharmacology professional societies to apply clinical pharmacological principles in the search for safe and efficacious treatments for COVID‐19.^10^

## COMPETING INTERESTS

M.G.D, R.K and C.R.R. work for Certara, a consulting firm in integrated drug development, and have directly consulted with a variety of not‐for‐profit global health organizations, biotechnology, and pharmaceutical companies, and governments with an interest in medical countermeasures against respiratory virus infections. A.G. is a PhD student funded by Roche.

## CONTRIBUTORS

Authors contributed to the following elements of this submission: (1) study concept, analysis planning and interpretation (M.G.D., R.K, C.R.R.); (2)Analyses (M.G.D., A.G.); (3) Writing of first draft, important intellectual revision and final manuscript writing and submission, response to reviewers and approval of completed manuscript (M.G.D., R.K., A.G., C.R.R.).

## Supporting information

**Supporting Information Figure S1** Equivalent drug effect sizes for inhibitory and stimulatory processes in the model

**Supporting Information Figure S2** Comparisons of all target treatments by treatment initiation time, endpoint and summed drug effect

**Supporting Information Figure S3** Example combination treatments assuming intervention 6 days before viral peak

**Supporting Information Figure S4** Example combination treatments assuming intervention 6 days after viral peak

**Supporting Information Data S1** Script S1. R script reproducing the analysis

## Data Availability

The R script reproducing the analyses presented are included in the Supporting Information.
