# Supplementary figures and images for: Model‐informed drug repurposing: Viral kinetic modelling to prioritize rational drug combinations for COVID‐19
Source: Br J Clin Pharmacol. 2020 Aug 5;87(9):3439–50. doi: 10.1111/bcp.14486 (PMC8451752; doi:10.1111/bcp.14486)

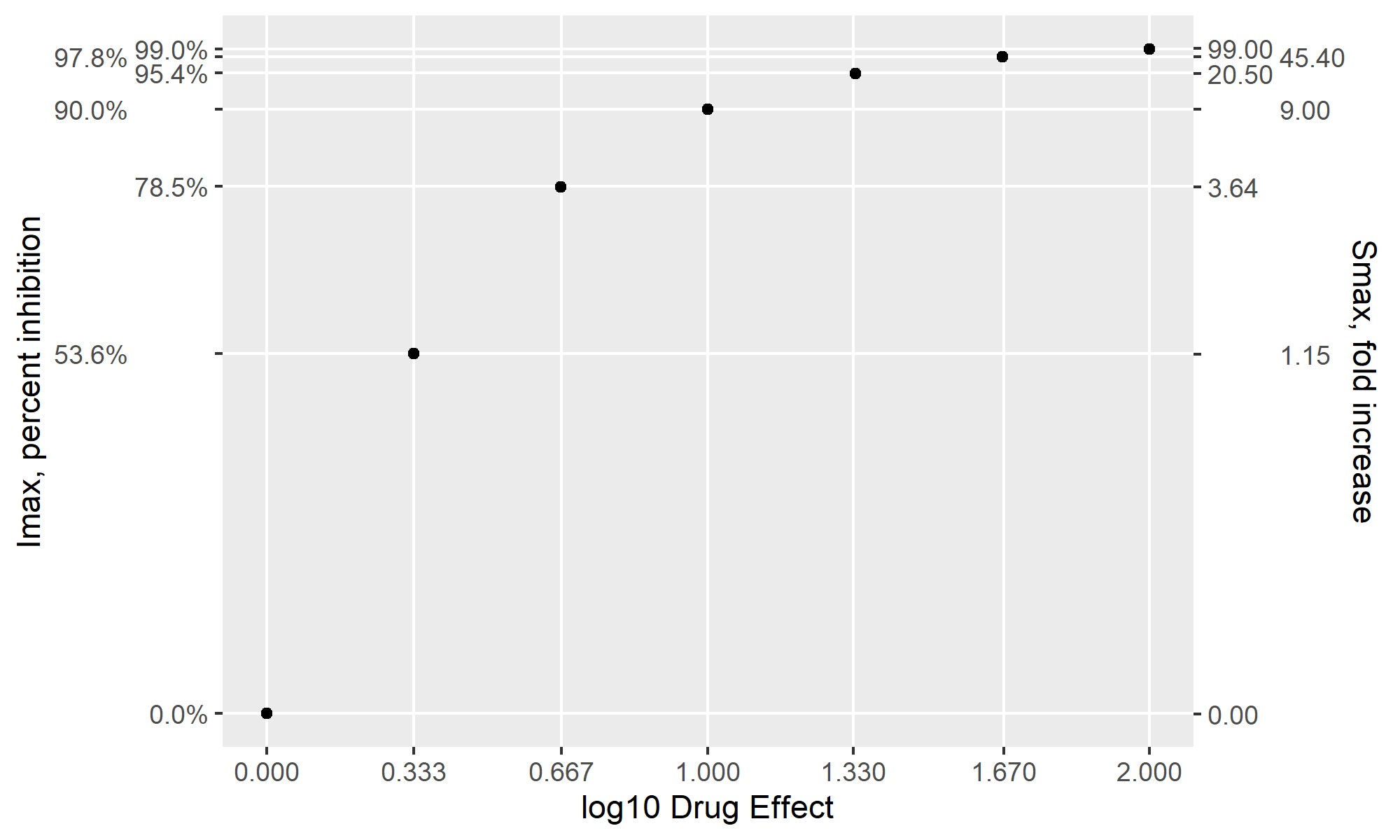

Supplement: Supplementary file 1 — Supporting Information Figure S1 Equivalent drug effect sizes for inhibitory and stimulatory processes in the model [file BCP-87-3439-s005.png]

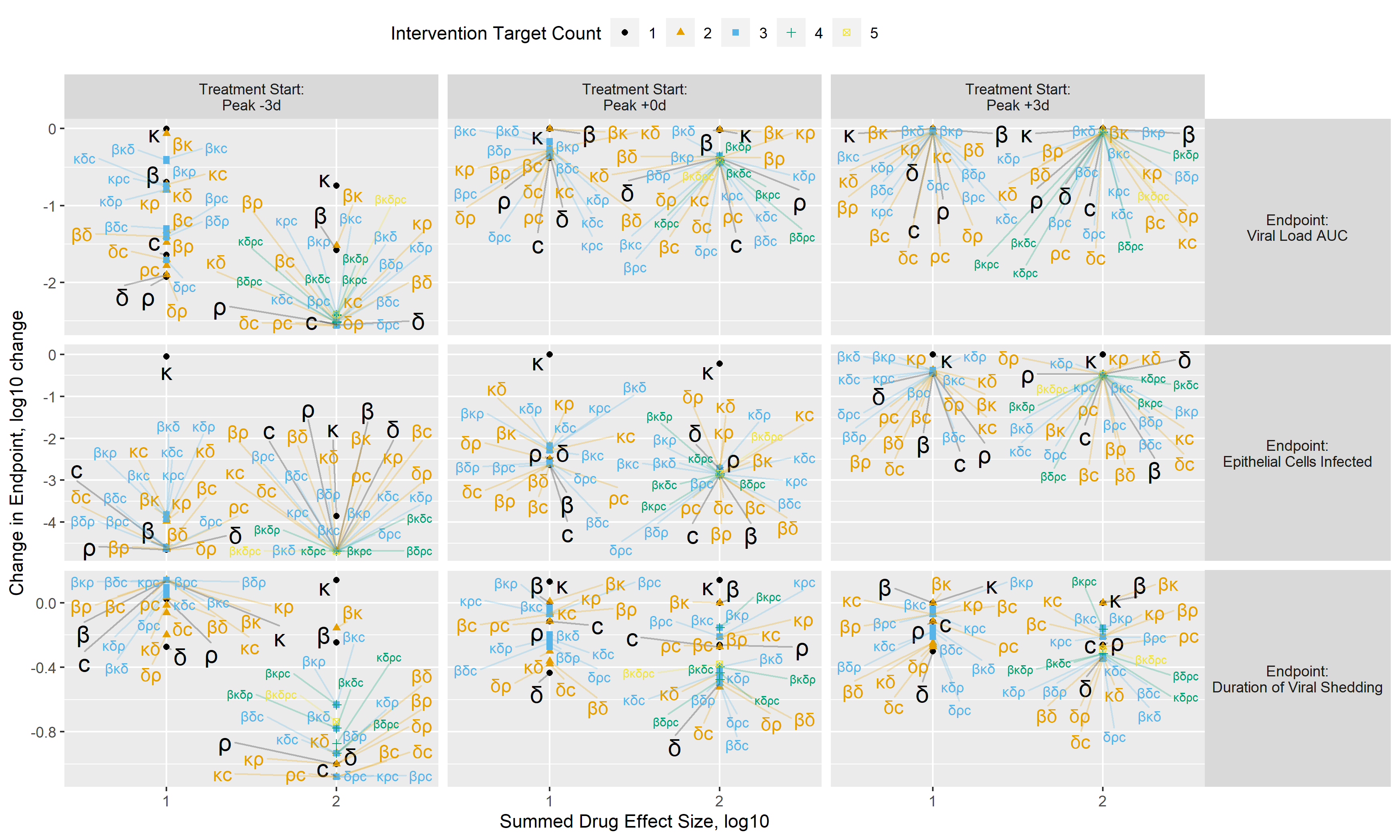

Supplement: Supplementary file 2 — Supporting Information Figure S2 Comparisons of all target treatments by treatment initiation time, endpoint and summed drug effect [file BCP-87-3439-s001.png]

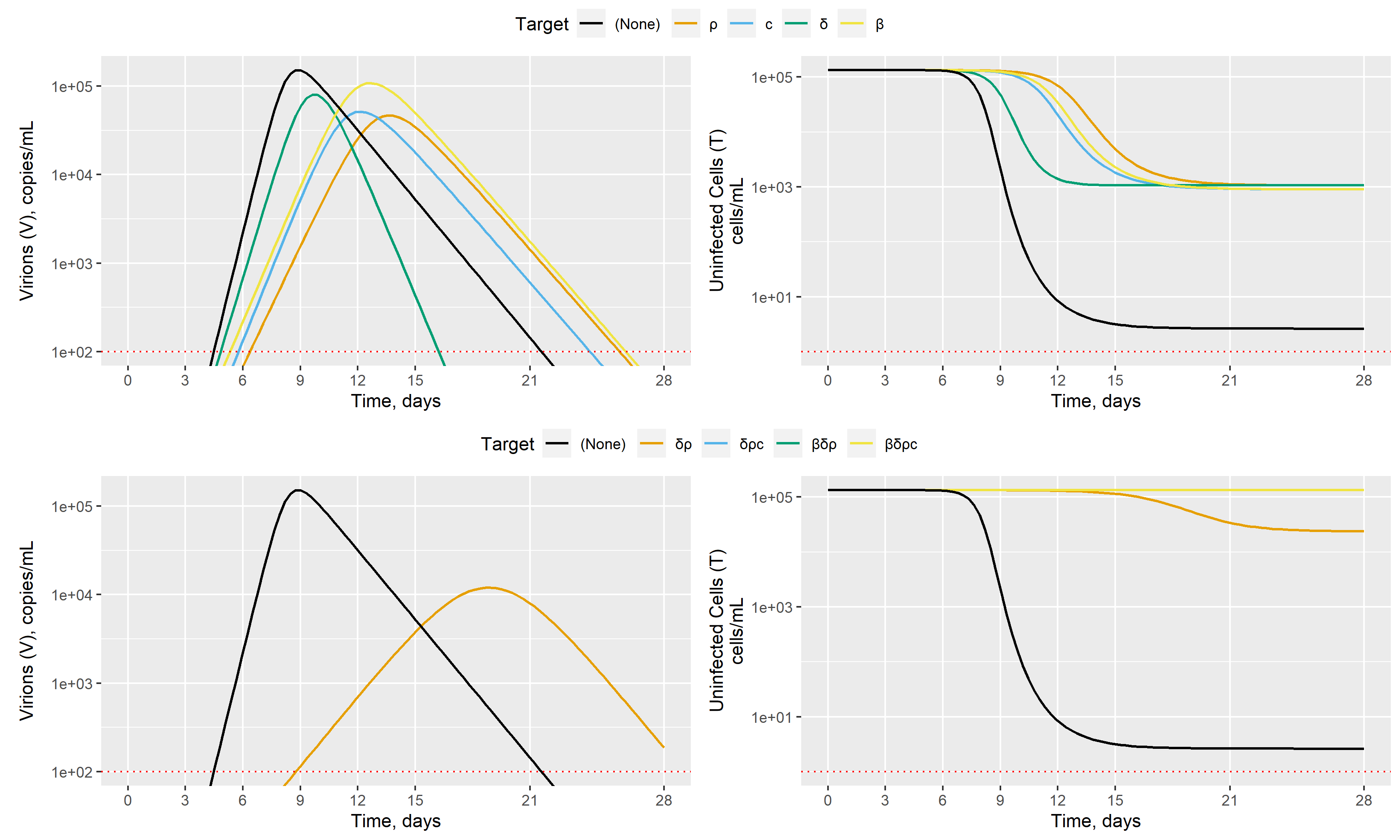

Supplement: Supplementary file 3 — Supporting Information Figure S3 Example combination treatments assuming intervention 6 days before viral peak [file BCP-87-3439-s003.png]

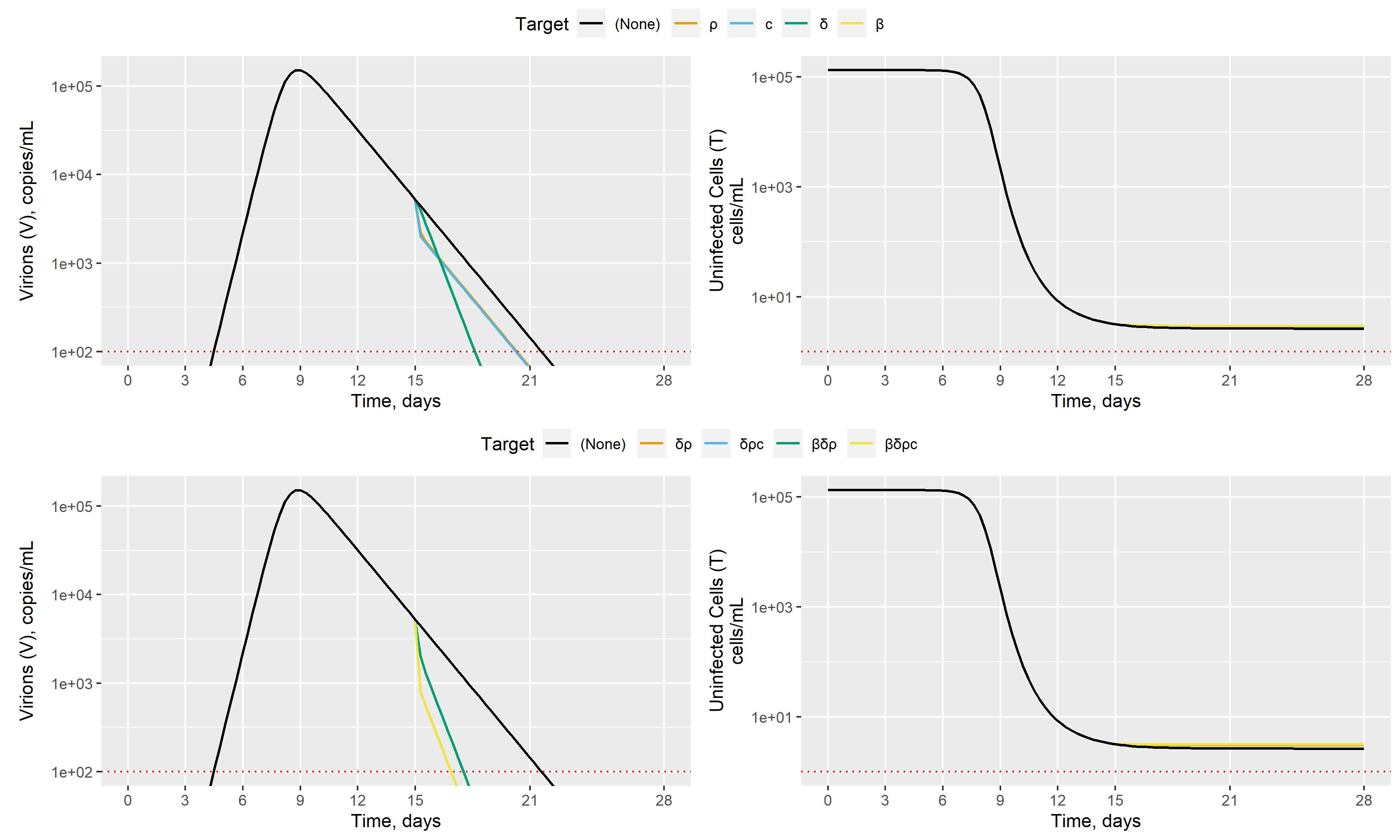

Supplement: Supplementary file 4 — Supporting Information Figure S4 Example combination treatments assuming intervention 6 days after viral peak [file BCP-87-3439-s002.png]
