# Supplementary material for: Model‐informed drug repurposing: Viral kinetic modelling to prioritize rational drug combinations for COVID‐19
Source: Br J Clin Pharmacol. 2020 Aug 5;87(9):3439–50. doi: 10.1111/bcp.14486 (PMC8451752; doi:10.1111/bcp.14486)
Supplement: Supplementary file 5 — Supporting Information Data S1 Script S1. R script reproducing the analysis [file BCP-87-3439-s004.docx]

**Supplemental Material**

**Supplemental Figure 1.** Equivalent drug effect sizes for inhibitory and stimulatory processes in the model.


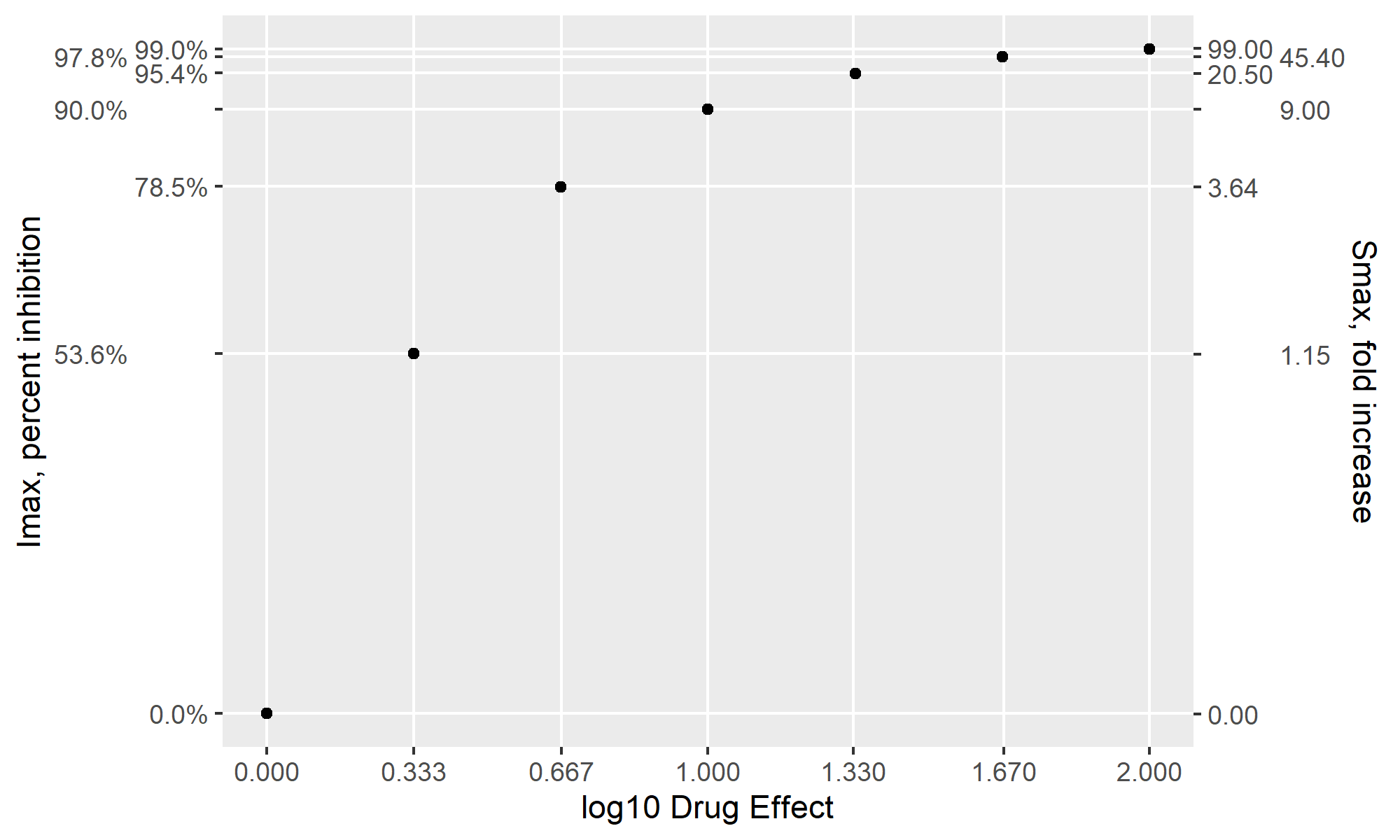


**Supplemental Figure 2.** Comparisons of all target treatments by treatment initiation time, endpoint and summed drug effect.


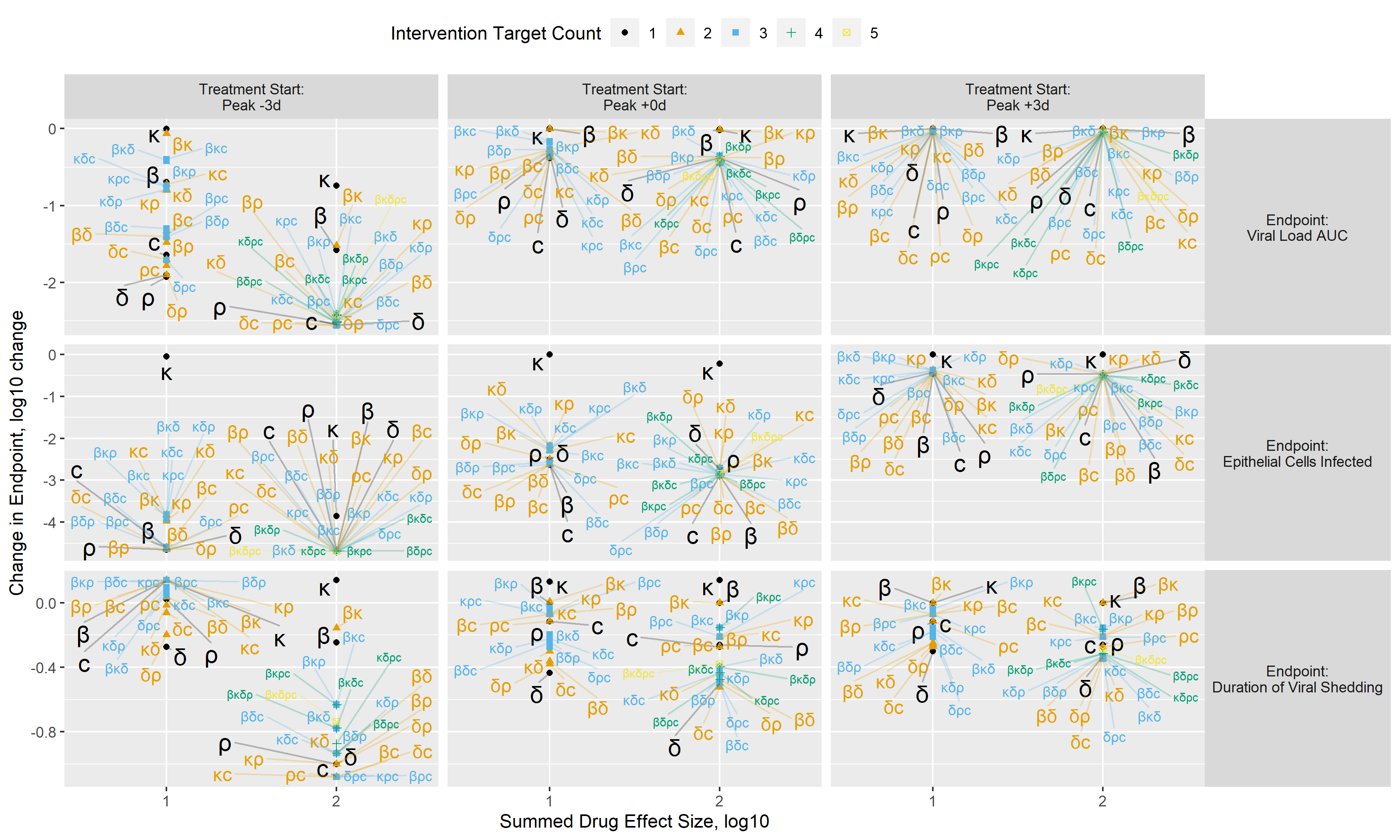


Treatment initiation is shown by column relative to expected viral load peak; endpoint is shown by row; Change in endpoint relative to the no-treatment control is reported on the y-axis as log10( treatment outcome metric / no-treatment outcome metric ) with negative values representing improvements relative to the no-treatment control; number of targets in each intervention is shown by color, size and shape; each intervention is plotted by its summed drug effect on the x-axis; labels of the treatment targets appear with lines to connect them to the specific point on the diagram.

**Supplemental Figure 3.** Example combination treatments assuming intervention six days before Viral Peak.


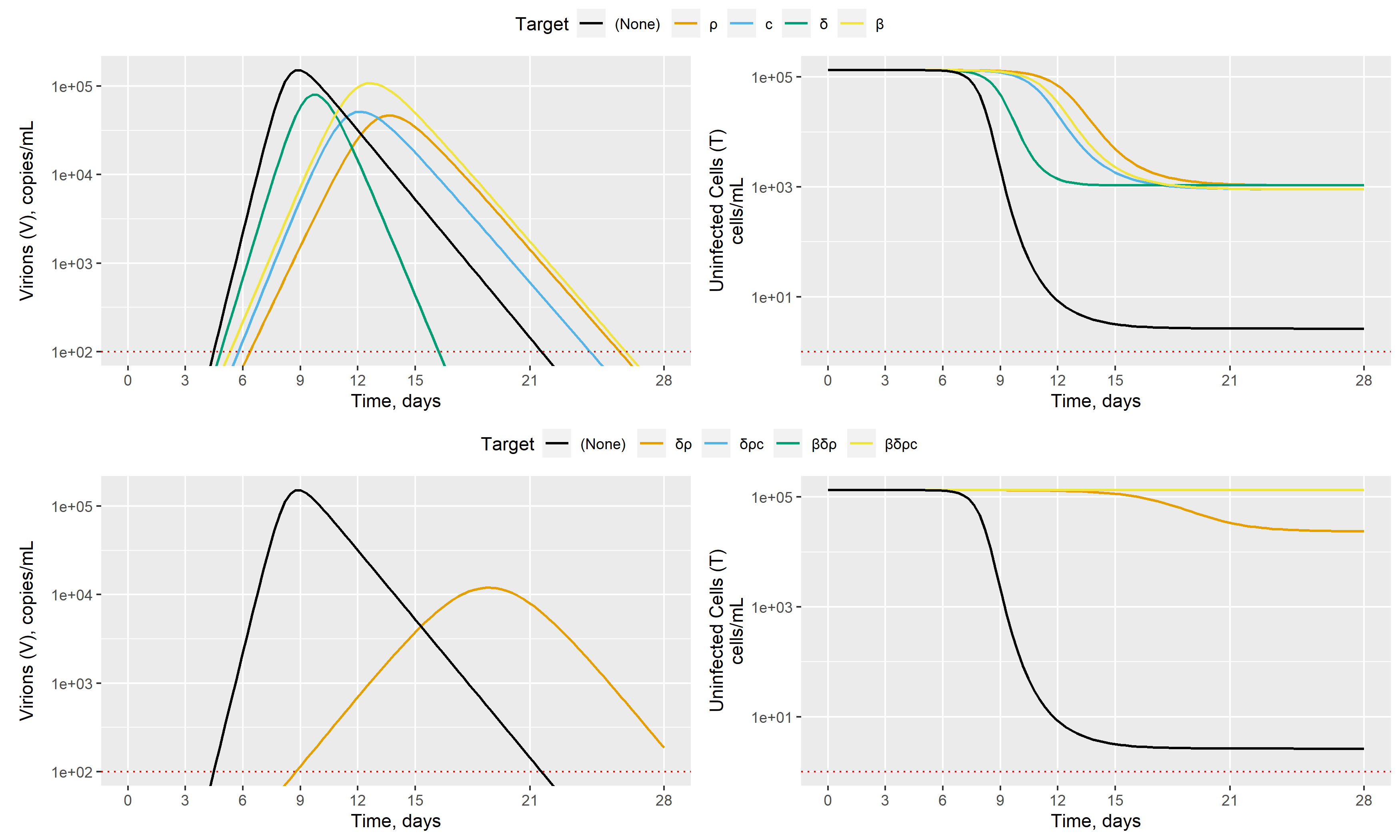


**Supplemental Figure 4.** Example combination treatments assuming intervention six days after Viral Peak.


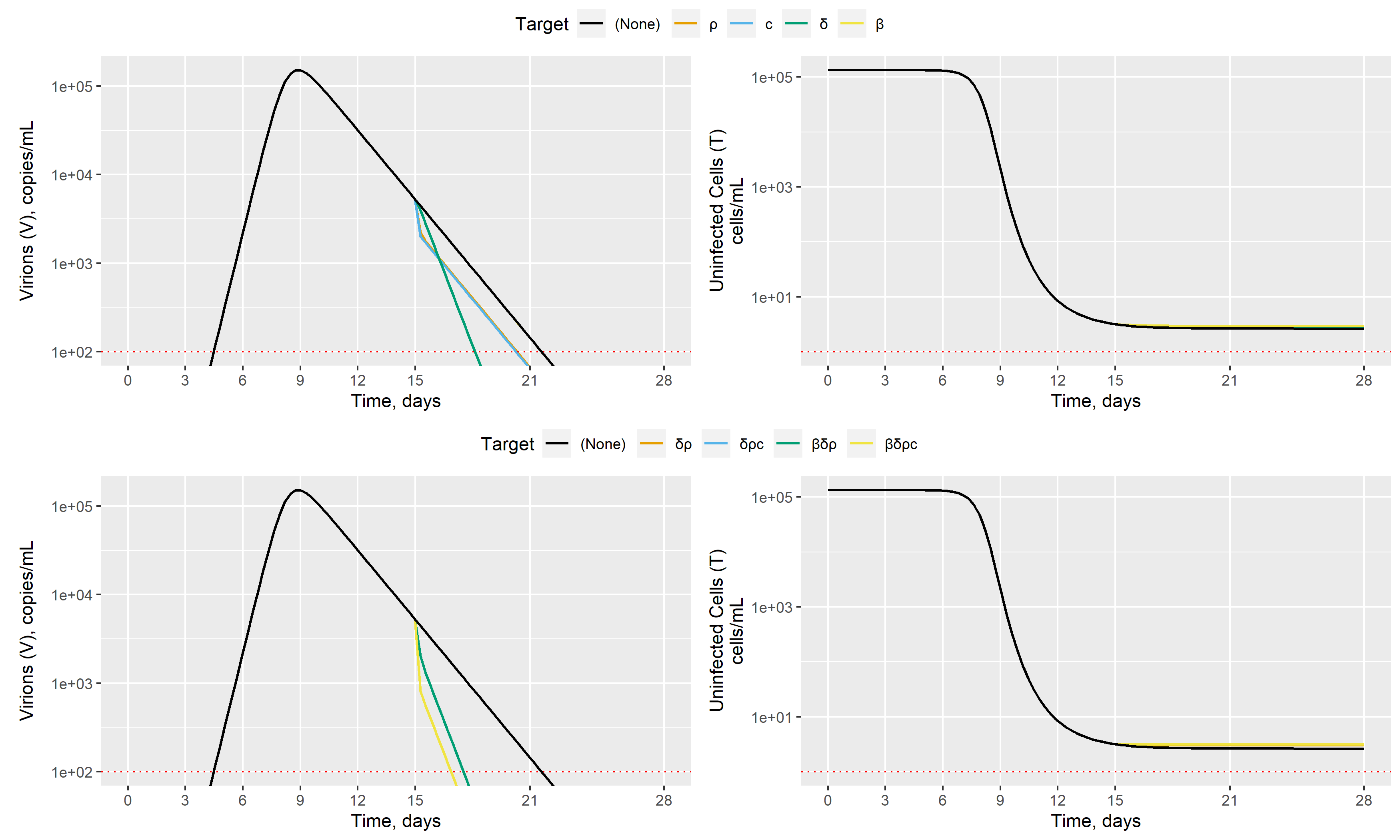


**Supplemental Script 1.** R script reproducing the analysis

# modeling

library(RxODE)

library(caTools)

# data

library(dplyr)

library(magrittr)

library(forcats)

library(readr)

library(tidyr)

library(purrr)

library(stringr)

# plotting

library(ggplot2)

library(ggthemes)

library(scales)

library(ggpubr)

library(ggrepel)

# helpers

write_png = function(p, file, h=6, w=5/3*h) {

ggsave(file, plot=p, width=w, height=h, units="in", dpi=300)

}

drop_lists = function(df) {

df %>% select_if( ~(! is_list(.)) )

}

dir.create("Figures")

dir.create("Tables")

# ceate a model specification

ode = "

# Viral dynamics was fitted using a target cell limited model with an eclipse phase.

# Goncalves 2020, Equation 1

# The model considers three populations of cells: target cells, T, infected cells in the eclipse phase, I1, and productively infected cells, I2.

# Given the timescale of the infection, we neglect target cell proliferation and natural death, and focux on the process of cell depletion by virus infection.

# We assume target cells, T, become infected virions, V, with rate constant b.

# Infection can be inhibited by drugging, up to a fractional inhibition, Iinh.

d/dt(Iinh) = 0;

Infection = b*V*T * (1-Iinh);

d/dt(T) = - Infection;

# After an average time of 1/k, infected cells in the eclipse phase, I1, convert to productively infected cells, I2.

# Conversion can be inhibited by drugging, up to a fractional inhibition, Cinh.

d/dt(Cinh) = 0;

Conversion = k*I1 * (1-Cinh);

d/dt(I1) = Infection - Conversion;

# Productively infected cells, I2, die with a per capita rate d.

# Death can be promoted by drugging, up to a fold increase, Dinc.

d/dt(Dinc) = 0;

Death = d*I2 * (1+Dinc);

d/dt(I2) = Conversion - Death;

# Virions are released from productively infected cells I2 at rate p per cell and are killed in circulation at per capita rate c or lost by infecting a target cell.

# Release can be inhibited by drugging, up to a fractional inhibition, Rinh.

d/dt(Rinh) = 0;

# Kill can be promoted by drugging, up to a fold increase, Kinc.

d/dt(Kinc) = 0;

Release = p*I2 * (1-Rinh);

Kill = c*V * (1+Kinc);

d/dt(V) = Release - Kill - Infection;

# Based on this model, the basic reproduction number, R0, the average number of cells infected by a single infected cell at the beginning of the infection, is:

# Equation 2

R0 = p*b*T0/d/(c+b*T0);

V(0) = V0;

T(0) = T0;

"

model.obj = RxODE(model = ode)

print(model.obj)

# natural history of the disease

# Goncalves 2020

theta.Goncalves = c(

### viral model

V0 = 1e-1, # #/mL

T0 = 4e8*0.01/30, # #/mL

k = 3, # 1/d

d = 0.60, # 1/d

R0 = 8.6, # (unitless)

p = 22.71, # 1/d BUT #/d in the manuscript

c = 10 # 1/d

) %>% signif(4)

pT0 = with(as.list(theta.Goncalves), p*T0)

print(pT0) # 3.02e6 #/d

b = with(as.list(theta.Goncalves), R0*d*c/(T0*(p-(R0*d))) )

print(b) # 2.21e-5 mL/#/day

theta.Goncalves[["b"]] = b

# model parameters

model.theta = theta.Goncalves

# and a simulation wrapper

Simulate = function(Events) {

rxSolve(

object=model.obj,

params=model.theta,

events=Events,

seed=8675309 # don't lose my number

) %>%

drop_units %>%

as_tibble %>%

select(

time,

V, I1, I2, T,

Iinh, Cinh, Dinc, Rinh, Kinc

) %>%

mutate(

Tn=T/T[1], # fraction remaining

Vn=V/V[1] # fraction remaining

)

}

# create the model input as an EventTable,

# including dosing and observation (sampling) events

# https://nlmixrdevelopment.github.io/RxODE/articles/RxODE-event-table.html

Sampling.Events = function(Days=4*7, len=1000) {

et(time.units="days") %>% et(seq(0, Days, len=len))

}

Natural.History = Sampling.Events()

Break.Times = function(Events) {

sampling.times = Events$get.sampling() %$% {range(time)}

treatment.times = NULL

if( !is.null(Events$get.dosing()) ) treatment.times = Events$get.dosing() %$% {time}

sort(unique(c(sampling.times, treatment.times)))

}

break.times = Natural.History %>% Break.Times

# units, etc.

Labels = tribble(

~Name, ~Unit, ~Label,

"time", "days", "Time",

# states

"V", "copies/mL", "Virions (V)",

"I1", "cells/mL", "Infected Eclipse Cells (I1)",

"I2", "cells/mL", "Infected Productive Cells (I2)",

"T", "cells/mL", "Uninfected Cells (T)"

) %>%

mutate( Label.Units = ifelse(nchar(Label)>12, sprintf("%s\n%s", Label, Unit), sprintf("%s, %s", Label, Unit)) ) %>%

mutate_if(is.character, as_factor)

Label = function(name) { Labels %>% filter(Name==name) %$% {Label} }

Label.and.Units = function(name) { Labels %>% filter(Name==name) %$% {Label.Units} }

Beta = "\U03B2"

Kappa = "\U03BA"

Delta = "\U03B4"

Rho = "\U03C1"

Cee = "c"

Plot = function(sim, y, x="time", breaks=break.times) {

stopifnot(length(x)==1)

gvars = sim %>% group_vars

if(length(gvars)==0) { gvars=NULL }

stopifnot(length(gvars)<2)

if(length(y) > 1) {

sim %>%

select(one_of(gvars, x, y)) %>%

gather(Name, Value, -one_of(gvars, x)) %>%

left_join(Labels, by="Name") %>%

ggplot +

aes_string(x=x, y="Value", group=gvars, color=gvars) +

geom_line() +

scale_x_continuous(breaks=breaks, minor_breaks=7*(0:52)) +

labs(

x=Labels %>% filter(Name==x) %$% Label.Units,

y=NULL

) +

facet_wrap(~Label.Units, scales="free_x") +

scale_color_colorblind()

} else {

sim %>%

ggplot +

aes_string(x=x, y=y, group=gvars, color=gvars) +

geom_line() +

scale_x_continuous(breaks=breaks, minor_breaks=7*(0:52)) +

labs(

x=Labels %>% filter(Name==x) %$% Label.Units,

y=Labels %>% filter(Name==y) %$% Label.Units

) +

scale_color_colorblind()

}

}

# simulate interventions

logC.to.Smax = function(lC) { 10^lC-1 }

logC.to.Imax = function(lC) { 1-1/10^lC }

Smax.to.logC = function(Sm) { log10(Sm+1) }

Imax.to.logC = function(Im) { -log10(1-Im) }

prom.inh.values = tibble(

# consider a range of log10 changes by half-log increments

logC = sort(unique(c(0, 1/3*c(1,2,3), 2/3*c(1,2,3), 5/3)))

) %>%

mutate(

# calculate Smax and Imax, so we have "fair" comparisons

Smax = logC %>% logC.to.Smax,

Imax = logC %>% logC.to.Imax

) %T>% print

prom.inh.values %>% mutate_all(signif, 3) %>% rename(`Log Effect`=logC) %>% write_csv('Tables/Effect Size Choices.csv')

p.eff = qplot(logC, Imax, data=prom.inh.values) +

scale_x_continuous(breaks=signif(unique(prom.inh.values$logC),3), minor_breaks=F) +

scale_y_continuous(

breaks=signif(unique(prom.inh.values$Imax),3), minor_breaks=F, labels=percent,

name="Imax, percent inhibition",

guide=guide_axis(n.dodge=2),

sec.axis=sec_axis(

trans=~logC.to.Smax(Imax.to.logC(.)),

breaks=signif(unique(prom.inh.values$Smax),3),

labels=waiver(),

name="Smax, fold increase",

guide=guide_axis(n.dodge=2)

)

) +

labs(x="log10 Drug Effect")

p.eff %>% write_png("Figures/Effect Size Choices.png", h=4)

# when do we hit peak virus?

Time.to.Peak = function(df) {

df %>% filter(V==max(V)) %$% {time} %>% round(1)

}

# natural history

study.duration = 28

Natural.History = Sampling.Events(study.duration, len=100)

sim.NH = Natural.History %>% Simulate

V.peak = sim.NH %>% Time.to.Peak %>% round # days

# simulations

effect.choices = prom.inh.values$logC

effect.N.max = 5

effect.N.min = 1

effect.sum.max = max(effect.choices)

effect.sum.min = min(effect.choices[effect.choices>0])

start.choices = V.peak + c(-6, -3, 0, 3, 6)

break.times = c(0, start.choices, 21, study.duration)

# all the permutations

Sim.Cases = crossing(

Infection = effect.choices,

Conversion = effect.choices,

Death = effect.choices,

Release = effect.choices,

Kill = effect.choices,

Start = start.choices

) %>%

mutate(

Eff.Total = Infection + Conversion + Death + Release + Kill,

Eff.N = (Infection>0) + (Conversion>0) + (Death>0) + (Release>0) + (Kill>0)

) %>%

filter(

# Natural History comparator

(Infection + Conversion + Death + Release + Kill == 0) | # OR

(

# treatments are between min and max effect

between(Eff.Total, effect.sum.min, effect.sum.max) &

# treatments are between min and max count

between(Eff.N, effect.N.min, effect.N.max)

)

) %>%

distinct %>%

mutate(

Sim=1:n()

) %>%

nest( Tx=c(-Sim) )

Intervention.Events = function(Events, Intervention, Value, Start, Stop, Onset, Offset) {

Events %>%

# adding a tiny onset and offset time, because I'm getting bizzare failures with mixtures of zero

# onset and offset values

et(dosing.to=Intervention, amt=Value, time=Start, rate=Value/(Onset+1e-5)) %>%

et(dosing.to=Intervention, amt=-Value, time=Stop+Offset, rate=Value/(Offset+1e-5))

}

Treatment.Events = function(df, events=Natural.History, Stop=study.duration) {

if( df$Infection>0 ) events %<>% Intervention.Events("Iinh", logC.to.Imax(df$Infection), df$Start, Stop, 0, 0)

if( df$Conversion>0 ) events %<>% Intervention.Events("Cinh", logC.to.Imax(df$Conversion), df$Start, Stop, 0, 0)

if( df$Death>0 ) events %<>% Intervention.Events("Dinc", logC.to.Smax(df$Death), df$Start, Stop, 0, 0)

if( df$Release>0 ) events %<>% Intervention.Events("Rinh", logC.to.Imax(df$Release), df$Start, Stop, 0, 0)

if( df$Kill>0 ) events %<>% Intervention.Events("Kinc", logC.to.Smax(df$Kill), df$Start, Stop, 0, 0)

events

}

Compute.Outcomes = function(df) {

df %>%

summarise(

T_min = min(T),

V_auc = trapz(time, V),

V_dur = diff(range(time[V>100]))

)

}

Simulations = Sim.Cases %>%

mutate(

Events = map(Tx, Treatment.Events),

Sims = map(Events, Simulate),

Out = map(Sims, Compute.Outcomes)

)

### compare overall

Out = Simulations %>%

unnest(c(Tx)) %>%

unnest(c(Out)) %>%

drop_lists

# to what?

ref.simn = Out %>% filter(Infection + Conversion + Death + Release + Kill == 0) %$% Sim[1]

ref.sim = Simulations$Sims[[ref.simn]]

ref.label = "(None)"

# save it

ggarrange(

plotlist=list(

Plot(ref.sim, "T") + scale_y_log10() + scale_x_continuous(breaks=break.times, minor_breaks=F) + coord_cartesian(ylim=c(1, NA)),

Plot(ref.sim, "I1") + scale_y_log10() + scale_x_continuous(breaks=break.times, minor_breaks=F) + coord_cartesian(ylim=c(1, NA)),

Plot(ref.sim, "I2") + scale_y_log10() + scale_x_continuous(breaks=break.times, minor_breaks=F) + coord_cartesian(ylim=c(1, NA)),

Plot(ref.sim, "V") + scale_y_log10() + scale_x_continuous(breaks=break.times, minor_breaks=F) + coord_cartesian(ylim=c(100, NA))

),

align="hv", ncol=2, nrow=2

) %>% write_png("Figures/Natural History.png", 7)

p.super = Plot(ref.sim, c("T","V")) +

facet_null() +

scale_y_log10() +

aes(color=Label.Units) +

coord_cartesian(ylim=c(1,NA), xlim=c(0,21)) +

theme(legend.pos="top") +

geom_hline(yintercept=c(100), color="red", linetype="dashed") +

geom_vline(xintercept=c(6, 9, 12), color="blue", linetype="dashed", size=1) +

labs(color="Endpoint")

p.super %>% write_png("Figures/Natural History Timeline.png", w=14/1.5, h=6/1.5)

Comp = Out %>%

mutate(

T_min = log10(T_min[1]/T_min), # flip sign

V_auc = log10(V_auc/V_auc[1]),

V_dur = log10(V_dur/V_dur[1])

) %>%

gather( Endpoint, Value, T_min:V_dur ) %>%

mutate(

Value = ifelse(is.nan(Value) & Endpoint=="V_dur", -1.5, Value), # virus never is >LLOQ; scores well

End = Endpoint,

`Treatment Start` = factor(Start, levels=start.choices, labels=sprintf("Peak %+dd", start.choices-V.peak)),

`Endpoint` = factor(Endpoint, levels=c("V_auc","T_min","V_dur"), labels=c("Viral Load AUC","Epithelial Cells Infected","Duration of Viral Shedding")),

Eff.Total = signif(Eff.Total, 3),

`Total Effect` = factor(Eff.Total, levels=sort(unique(Eff.Total)),

labels=sprintf("%s log10\n%s %%\n%s fold",signif(sort(unique(Eff.Total)),3),signif(100*logC.to.Imax(sort(unique(Eff.Total))),3),signif(logC.to.Smax(sort(unique(Eff.Total))),3))),

Effect = paste0(

ifelse(Infection>0, Beta, ""),

ifelse(Conversion>0, Kappa, ""),

ifelse(Death>0, Delta, ""),

ifelse(Release>0, Rho, ""),

ifelse(Kill>0, Cee, "")

) %>% factor

) %>%

filter(Eff.N>0)

Comp %>% write_excel_csv("Tables/Comparison Table of Outcomes.csv")

awful_labeller_syntax = function(...) { label_both(..., sep=":\n") }

p.Comp.cont = ggplot(Comp) +

aes(x=Eff.Total, y=Value, color=factor(Eff.N), shape=factor(Eff.N)) +

facet_grid(`Endpoint`~`Treatment Start`, scales="free_y", labeller=awful_labeller_syntax) +

scale_color_colorblind() +

geom_smooth(aes(group=factor(Eff.N)), se=F, span=1.2) +

geom_point(alpha=0.2, position=position_jitter(width=0.1, height=0)) +

labs(x="Summed Drug Effect Size, log10", y="Change in Endpoint, log10 change", color="Intervention Target Count", shape="Intervention Target Count") +

theme(legend.pos="top") +

geom_hline(yintercept=0, linetype="dashed", color="red") +

scale_x_continuous(breaks=signif(unique(Comp$Eff.Total),3), minor_breaks=F, guide=guide_axis(n.dodge=2)) +

theme(strip.text.y=element_text(angle=0))

print(p.Comp.cont)

p.Comp.cont %>% write_png("Figures/Comparison Overview - continuous Effect scale.png")

p.Comp.cat = ggplot(Comp) +

aes(x=factor(Eff.Total), y=Value, color=factor(Eff.N), shape=factor(Eff.N)) +

facet_grid(`Endpoint`~`Treatment Start`, scales="free_y", labeller=awful_labeller_syntax) +

scale_color_colorblind() +

geom_point(alpha=0.1, position=position_jitter(width=0.3, height=0)) +

stat_summary(fun.data="median_hilow", geom="pointrange", mapping=aes(group=interaction(Eff.N, Eff.Total)), position=position_dodge2(width=0.75), shape=15) +

labs(x="Summed Drug Effect Size, log10", y="Change in Endpoint, log10 change", color="Intervention Target Count", shape="Intervention Target Count") +

theme(legend.pos="top") +

geom_hline(yintercept=0, linetype="dashed", color="red") +

scale_x_discrete(breaks=signif(unique(Comp$Eff.Total),3), guide=guide_axis(n.dodge=2)) +

theme(strip.text.y=element_text(angle=0))

print(p.Comp.cat)

p.Comp.cat %>% write_png("Figures/Comparison Overview - categorical Effect scale.png", 7)

# all of the options

cand1 = c(Beta, Kappa, Delta, Rho, Cee)

cand2=combn(c(Beta, Kappa, Delta, Rho, Cee), 2, simplify=F) %>% lapply(paste0, collapse="") %>% unlist

cand3=combn(c(Beta, Kappa, Delta, Rho, Cee), 3, simplify=F) %>% lapply(paste0, collapse="") %>% unlist

cand4=combn(c(Beta, Kappa, Delta, Rho, Cee), 4, simplify=F) %>% lapply(paste0, collapse="") %>% unlist

cand5=combn(c(Beta, Kappa, Delta, Rho, Cee), 5, simplify=F) %>% lapply(paste0, collapse="") %>% unlist

candidates=c(cand1, cand2, cand3, cand4, cand5)

efft = c(1,2)

srt = V.peak + c(-3, 0, 3)

Candidate = Comp %>%

filter(

Effect %in% candidates,

Eff.Total %in% efft,

Start %in% srt,

) %>%

mutate(

Effect=factor(Effect, levels=candidates)

) %>%

group_by(Start, End, Eff.Total, Effect) %>%

arrange(Value) %>% # take the best performer for this intervention

slice(1) %>%

ungroup %>%

transmute(Start, End, Eff.Total, # Effect, Value,

Result=signif(Value,3),

Effect

) %>%

spread(Effect, Result) %T>% print(n=50)

p.Cand = Candidate %>%

gather(Effect, Value, -Start, -End, -Eff.Total) %>%

mutate(

`Treatment Start` = factor(Start, levels=start.choices, labels=sprintf("Peak %+dd", start.choices-V.peak)),

Endpoint = factor(End, levels=c("V_auc","T_min","V_dur"), labels=c("Viral Load AUC","Epithelial Cells Infected","Duration of Viral Shedding")),

Effect=factor(Effect, levels=candidates),

Eff.N=str_length(Effect) %>% as_factor,

Eff.Total=Eff.Total %>% as_factor

) %>%

ggplot +

aes(x=factor(Eff.Total), y=Value, group=Effect) +

geom_point(aes(shape=Eff.N, color=Eff.N)) +

geom_text_repel(aes(label=Effect, color=Eff.N, size=Eff.N), min.segment.length=0, show.legend=F, segment.alpha=0.25) +

# facet_wrap(~Endpoint+`Treatment Start`, scales="free_y", ncol=3, labeller=label_both) +

facet_grid(Endpoint~`Treatment Start`, scales="free_y", labeller=awful_labeller_syntax) +

scale_color_colorblind() +

scale_size_manual(values=c("1"=rel(5), "2"=rel(4), "3"=rel(3), "4"=rel(2.5), "5"=rel(2.5))) +

theme(legend.pos="top") +

theme(strip.text.y=element_text(angle=0)) +

labs(

size="Intervention Target Count",

color="Intervention Target Count",

shape="Intervention Target Count",

x="Summed Drug Effect Size, log10",

y="Change in Endpoint, log10 change"

)

print(p.Cand)

p.Cand %>% write_png("Figures/Treatment Effect Comparison.png", 7)

(p.Cand %+% filter(p.Cand$data, Eff.N %in% c(1,2)) ) %>% write_png("Figures/Treatment Effect Comparison - 1 and 2.png", 7)

Plot.Range.vs.Field.by.Effect = function(Eff.ind, Start.time, Endpoint, Candidates) {

filter.y = function(df) {

df %>%

mutate_at(

vars(one_of(Endpoint)),

~ifelse(.<1e-7, 1e-7, .)

)

}

breaks=break.times

ylower = ifelse(Endpoint=="V", 100, 1)

No.Trt = Simulations %>%

unnest(c(Out)) %>%

unnest(c(Tx)) %>%

filter( Eff.Total == 0, Start == Start.time ) %>%

unnest(c(Sims)) %>%

filter.y %>%

select(time, no.Tx=one_of(Endpoint))

Field = Simulations %>%

unnest(c(Out)) %>%

unnest(c(Tx)) %>%

filter(

Start == Start.time,

Infection %in% c(0, Eff.ind),

Conversion %in% c(0, Eff.ind),

Death %in% c(0, Eff.ind),

Release %in% c(0, Eff.ind),

Kill %in% c(0, Eff.ind)

) %>%

unnest(c(Sims)) %>%

filter.y %>%

group_by(time) %>%

summarise_at(

vars(one_of(Endpoint)),

list(low=~quantile(.,0), mid=~quantile(.,0.5),high=~quantile(.,1))

) %>%

ungroup

Specifics = Simulations %>%

unnest(c(Out)) %>%

unnest(c(Tx)) %>%

mutate(

Effect = paste0(

ifelse(Infection>0, Beta, ""),

ifelse(Conversion>0, Kappa, ""),

ifelse(Death>0, Delta, ""),

ifelse(Release>0, Rho, ""),

ifelse(Kill>0, Cee, "")

)

) %>%

filter(

Start == Start.time,

Effect %in% Candidates,

Infection %in% c(0, Eff.ind),

Conversion %in% c(0, Eff.ind),

Death %in% c(0, Eff.ind),

Release %in% c(0, Eff.ind),

Kill %in% c(0, Eff.ind)

) %>%

unnest(c(Sims)) %>%

filter.y %>%

select(Effect, time, spec=one_of(Endpoint))

ggplot(No.Trt) +

aes(x=time) +

geom_line(data=Specifics, aes(y=spec, color=Effect), size=0.75) +

geom_line(aes(y=no.Tx, color="(None)"), size=0.75) +

scale_y_log10() +

coord_cartesian(ylim=c(ylower,NA)) +

scale_color_colorblind() +

scale_fill_colorblind() +

scale_x_continuous(breaks=breaks, minor_breaks=F) +

geom_hline(yintercept=ylower, linetype="dotted", color="red") +

labs(

color="Target",

x=Labels %>% filter(Name=="time") %$% Label.Units,

y=Labels %>% filter(Name==Endpoint) %$% Label.Units

)

}

Plot.Compares = function(c1, c2, toff, th=theme_grey()) {

ggarrange(

ggarrange(

plotlist=list(

Plot.Range.vs.Field.by.Effect(Eff.ind = effect.choices[2], Start.time = V.peak + toff, Endpoint = "V", Candidates = c1) + th,

Plot.Range.vs.Field.by.Effect(Eff.ind = effect.choices[2], Start.time = V.peak + toff, Endpoint = "T", Candidates = c1) + th

),

align="hv", ncol=2,

common.legend=T

),

ggarrange(

plotlist=list(

Plot.Range.vs.Field.by.Effect(Eff.ind = effect.choices[2], Start.time = V.peak + toff, Endpoint = "V", Candidates = c2) + th,

Plot.Range.vs.Field.by.Effect(Eff.ind = effect.choices[2], Start.time = V.peak + toff, Endpoint = "T", Candidates = c2) + th

),

align="hv", ncol=2,

common.legend=T

),

align="hv", ncol=1

)

}

cand1 = c(Beta, Delta, Rho, Cee)

cand234 = c(paste0(Delta, Rho), paste0(Beta, Delta, Rho), paste0(Delta, Rho, Cee), paste0(Beta, Delta, Rho, Cee))

p.tm6 = Plot.Compares(cand1, cand234, -6)

print(p.tm6)

p.tm6 %>% write_png("Figures/Example Plots - Peak-6.png", 7)

p.tm3 = Plot.Compares(cand1, cand234, -3)

print(p.tm3)

p.tm3 %>% write_png("Figures/Example Plots - Peak-3.png", 7)

p.t = Plot.Compares(cand1, cand234, 0)

print(p.t)

p.t %>% write_png("Figures/Example Plots - Peak.png", 7)

p.tp3 = Plot.Compares(cand1, cand234, +3)

print(p.tp3)

p.tp3 %>% write_png("Figures/Example Plots - Peak+3.png", 7)

p.tp6 = Plot.Compares(cand1, cand234, +6)

print(p.tp6)

p.tp6 %>% write_png("Figures/Example Plots - Peak+6.png", 7)

border = theme(panel.border=element_rect(colour="black", fill=NA, size=1))

p.shebang = ggarrange(

p.tm3 + border,

p.t + border,

p.tp6 + border,

labels=c("A. Treatment Start: Peak -3d","B. Treatment Start: Peak +0d","C. Treatment Start: Peak +3d"),

align="hv", ncol=1,

hjust=-.05

) + border

p.shebang

s=1.5

p.shebang %>% write_png("Figures/Example Plots - All.png", h=9*s, w=6.5*s)

save.image(file=paste0('simulations_', format(Sys.time(), "%d%b%Y"), '.Rdata'))
